# Supplementary material for: Identification of the KIF18A alpha-4 helix as a therapeutic target for chromosomally unstable tumor cells
Source: bioRxiv. 2023 Oct 18:2023.10.16.562576. Preprint. [Version 1] doi: 10.1101/2023.10.16.562576 (PMC10614886; doi:10.1101/2023.10.16.562576)
Supplement: Supplement 1 [file NIHPP2023.10.16.562576v1-supplement-1.pdf]

bioRxiv preprint doi: https://doi.org/10.1101/2023.10.16.562576; this version posted October 19, 2023. The copyright holder for this preprint (which was not certified by peer review) is the author/funder, who has granted bioRxiv a license to display the preprint in perpetuity. It is made available under aCC-BY 4.0 International license.

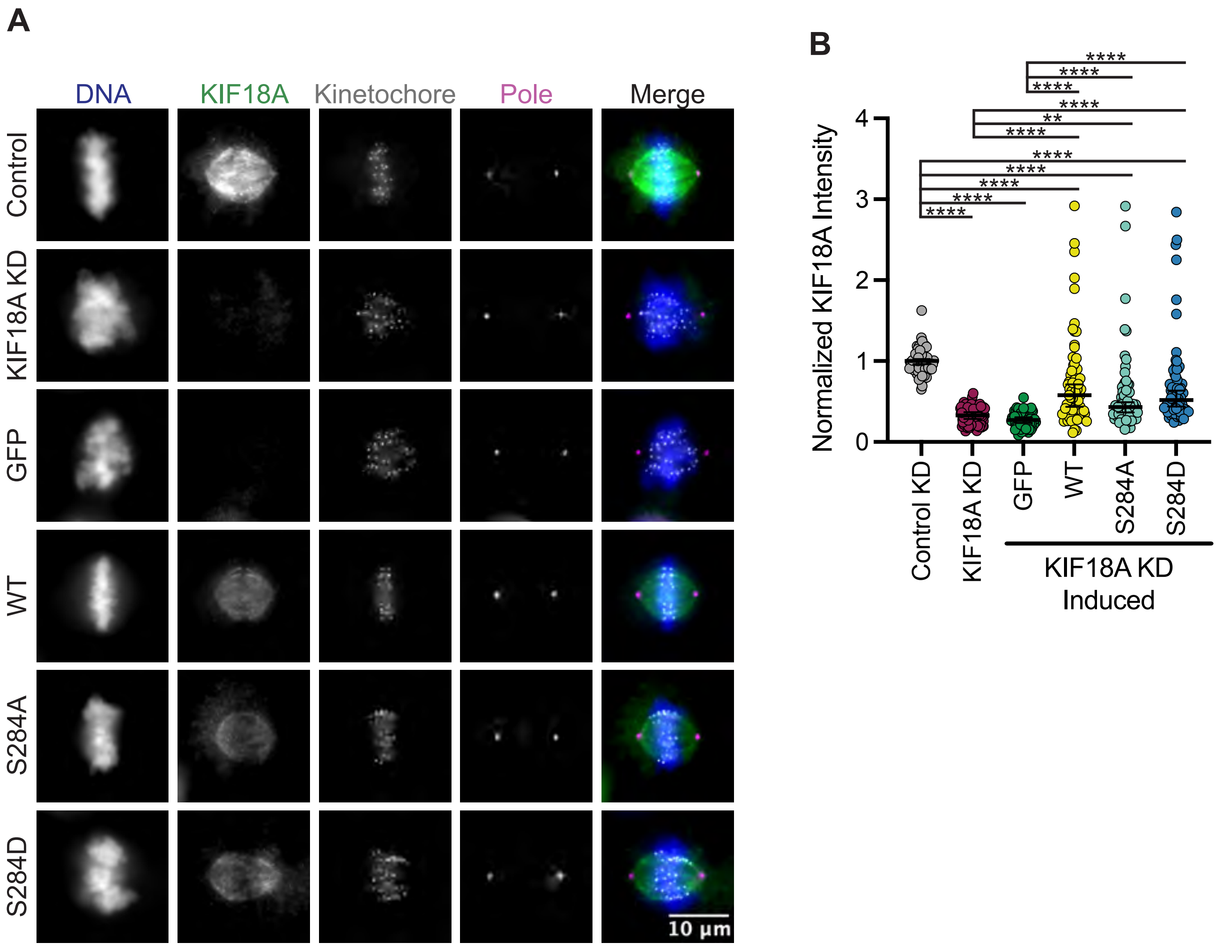

Figure S1: GFP-KIF18A S284 mutants are expressed at similar levels to wild type GFP-KIF18A. (A) Immunofluorescence images of HeLa Kyoto inducible cells fixed and stained 24 hours after endogenous KIF18A knockdown and induction of indicated GFP-KIF18A construct. Scale bar = 10 m. Text colors indicate pseudo color in merged image. From left to right DAPI/DNA, KIF18A antibody staining, CENP-C antibody staining, α-Tubulin antibody staining, merged image. **(B)** Quantification of KIF18A expression from KIF18A antibody staining. Fluorescence values were normalized to the mean control KIF18A intensity. Solid horizontal line indicates mean, vertical lines indicate standard deviation. Each dot represents a single cell. The total number of cells analyzed for each condition were control = 62, KIF18A KD = 80, GFP = 75, GFP-KIF18A WT = 68, GFP-S284A = 68, and GFP-S284D = 75. Data acquired from three experimental replicates. A one-way ANOVA with Tukey's test for multiple comparisons was run, P values: < 0.05 (*), < 0.01 (**), < 0.001 (***), <0.0001 (****). If no significance is indicated, result was not significant (> 0.05).

bioRxiv preprint doi: https://doi.org/10.1101/2023.10.16.562576; this version posted October 19, 2023. The copyright holder for this preprint (which was not certified by peer review) is the author/funder, who has granted bioRxiv a license to display the preprint in perpetuity. It is made available under aCC-BY 4.0 International license.

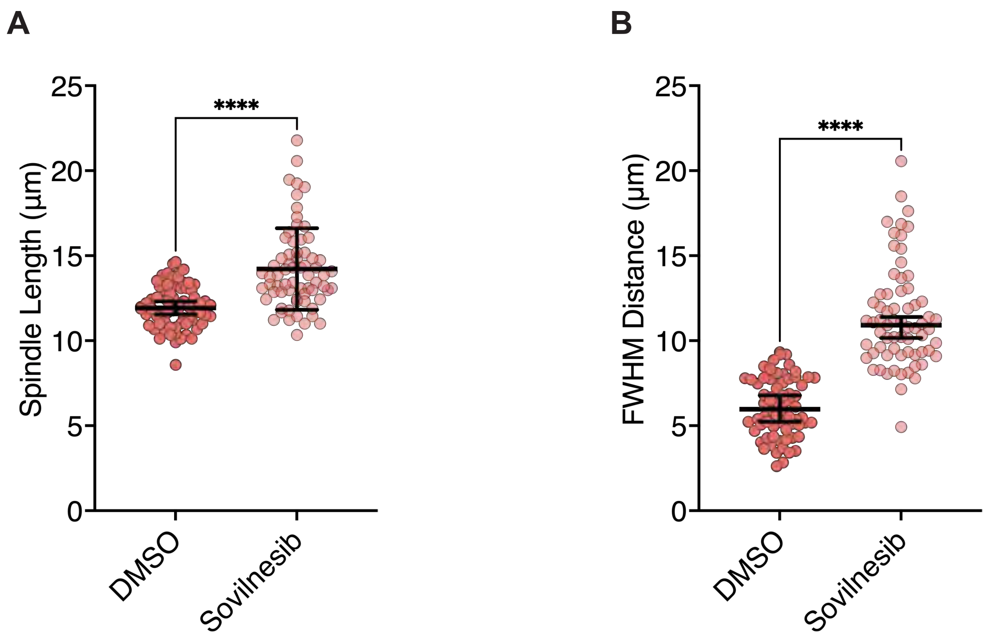

Figure S2: Sovilnesib mimics KIF18A knockdown phenotypes in MDA-MB-231 cells. Graphs of spindle lengths **(A)** and full-width at half maximum (FWHM) of centromere fluorescence distribution along the length of the spindle **(B)** measured in cells fixed and stained 24 hours after the indicated treatments. Each dot represents a single cell (N= 68 for DMSO, N=67 for Sovilnesib). Mean +/- standard deviation is displayed. Statistical results are shown for a Kruskal-Wallis with Dunn's Multiple Comparisons test. P value style: < 0.05 (*), < 0.01 (**), < 0.001 (***), <0.0001 (****). n.s. indicates not significant (> 0.05).

bioRxiv preprint doi: https://doi.org/10.1101/2023.10.16.562576; this version posted October 19, 2023. The copyright holder for this preprint (which was not certified by peer review) is the author/funder, who has granted bioRxiv a license to display the preprint in perpetuity. It is made available under aCC-BY 4.0 International license.

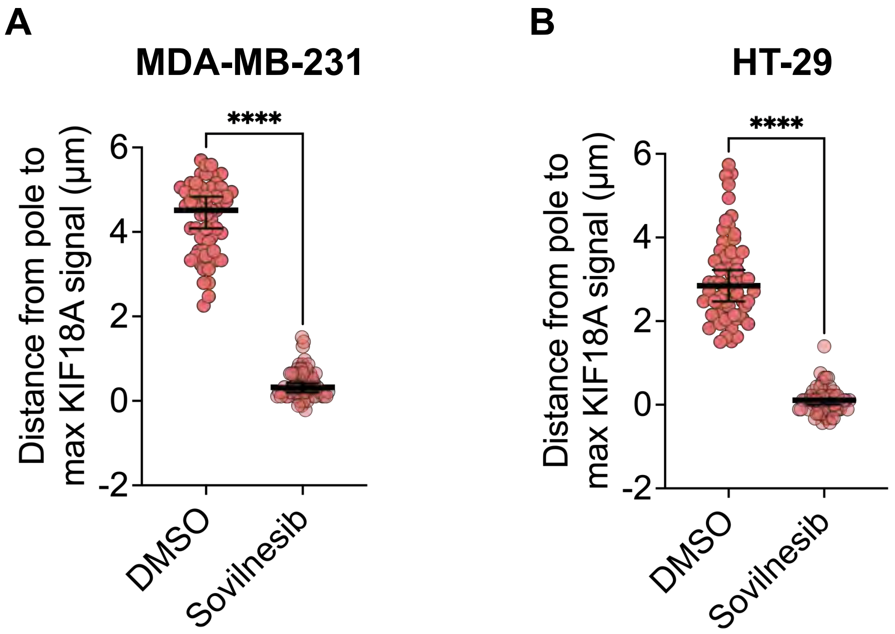

Figure S3: Sovilnesib disrupts microtubule plus-end localization of KIF18A. **(A-B)** Plots of distance from max KIF18A signal to the spindle pole derived from line scan analyses of KIF18A distribution in MDA-MB-231 **(A)** and HT-29 **(B)** cells. Each dot represents a single cell (MDA-MB-231: N=57 DMSO, N=60 Sovilnesib; HT-29: N=60 DMSO, N=60 Sovilnesib). Mean and SD are indicated by bars. Statistical results are shown for a Kruskal-Wallis with Dunn's Multiple Comparisons test. P value: < 0.05 (*), < 0.01 (**), < 0.001 (***), <0.0001 (****).

bioRxiv preprint doi: https://doi.org/10.1101/2023.10.16.562576; this version posted October 19, 2023. The copyright holder for this preprint (which was not certified by peer review) is the author/funder, who has granted bioRxiv a license to display the preprint in perpetuity. It is made available under aCC-BY 4.0 International license.

Figure S4.

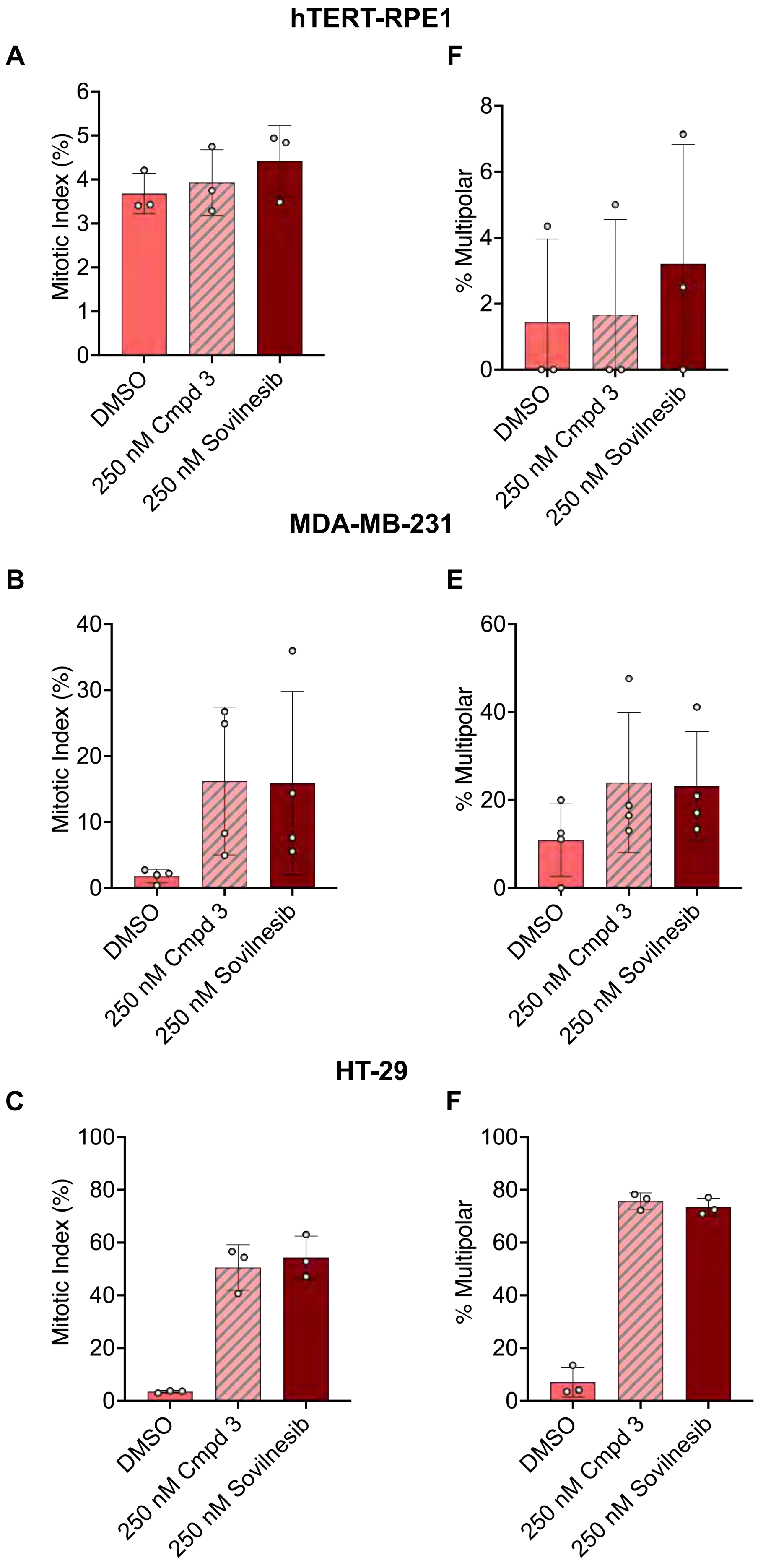

bioRxiv preprint doi: https://doi.org/10.1101/2023.10.16.562576; this version posted October 19, 2023. The copyright holder for this preprint (which was not certified by peer review) is the author/funder, who has granted bioRxiv a license to display the preprint in perpetuity. It is made available under aCC-BY 4.0 International license.

Figure S4: Sovilnesib treatment leads to mitotic arrest and multipolar spindles in chromosomally unstable breast and colorectal cancer cells. (A-C) Quantification of mitotic index (% of total cells in mitosis) in hTERT-RPE1 **(A)**, MDA-MB-231 **(B)**, and HT-29 cells **(C)** 24-hours after indicated treatments. Bars are mean +/- standard deviation. Each dot indicates an experimental replicate and data are from 3 independent experiments. **(D-F)** Quantification of multipolar spindles (% of total spindles) in hTERT-RPE1 **(D)**, MDA-MB-231, **(E)** and HT-29 cells **(F)** 24 hours after indicated treatments. Bars are mean +/- standard deviation. Each dot indicates an experimental replicate and data are from 3 independent experiments.

bioRxiv preprint doi: https://doi.org/10.1101/2023.10.16.562576; this version posted October 19, 2023. The copyright holder for this preprint (which was not certified by peer review) is the author/funder, who has granted bioRxiv a license to display the preprint in perpetuity. It is made available under aCC-BY 4.0 International license.

Figure S5.

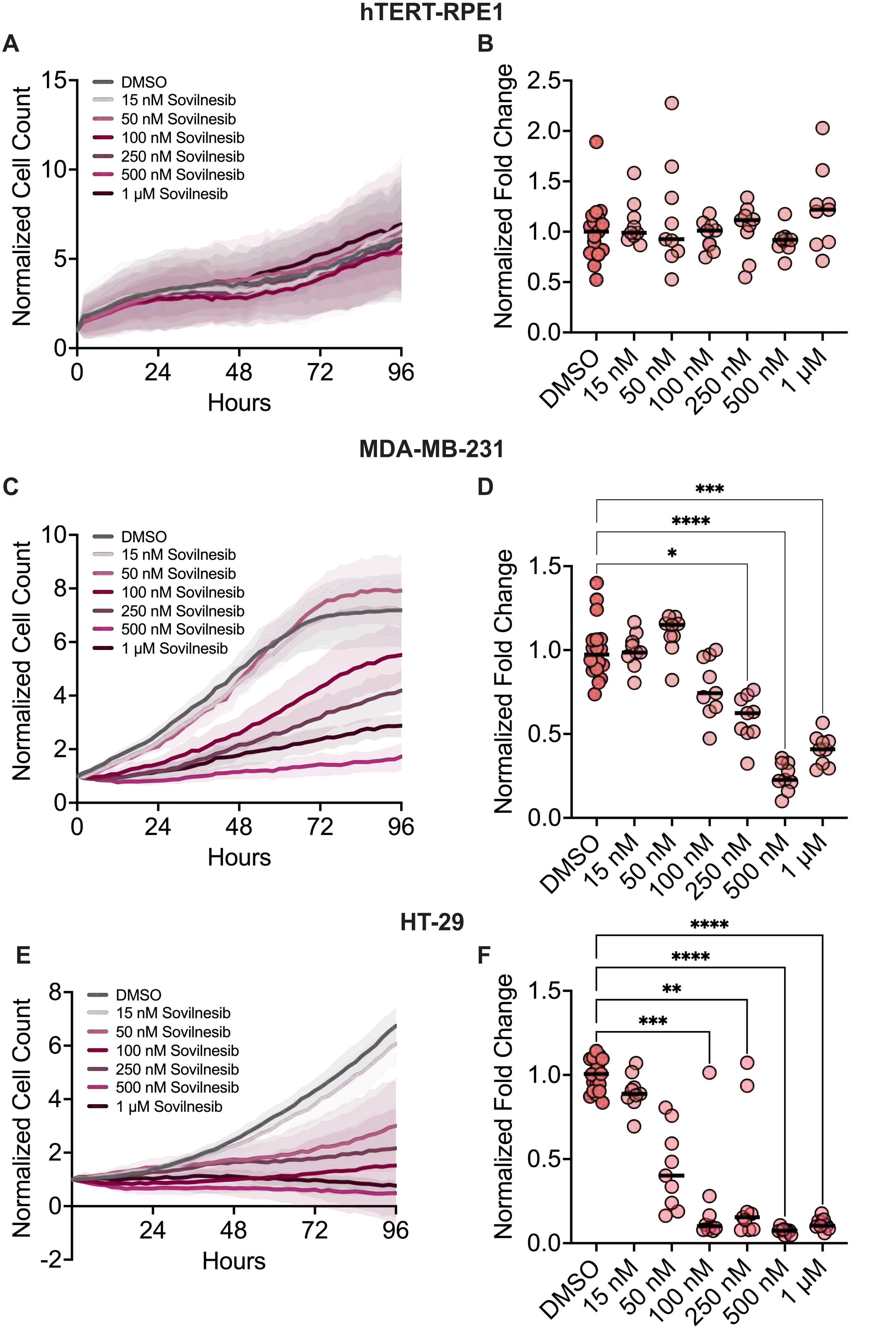

bioRxiv preprint doi: https://doi.org/10.1101/2023.10.16.562576; this version posted October 19, 2023. The copyright holder for this preprint (which was not certified by peer review) is the author/funder, who has granted bioRxiv a license to display the preprint in perpetuity. It is made available under aCC-BY 4.0 International license.

Figure S5: Sovilnesib reduces the proliferation of chromosomally unstable cells in a dose-dependent manner. (A) Plot of normalized cell count over time for cells treated with the indicated concentrations of Sovilnesib. Lines indicate mean and shaded area denotes SD. **(B)** Plot of normalized hTERT-RPE1 cell count (displayed as a % of DMSO control) as a function of Sovilnesib concentration. **(C)** Plot of normalized cell count over time for MDA-MB-231 cells treated with the indicated concentrations of Sovilnesib. Lines indicate mean and shaded area denotes SD. **(D)** Plot of normalized MDA-MB-231 cell count (displayed as a % of DMSO control) as a function of Sovilnesib concentration. **(E)** Plot of normalized cell count over time for HT-29 cells treated with the indicated concentrations of Sovilnesib. Lines indicate mean and shaded area denotes SD. **(F)** Plot of normalized HT-29 cell count (displayed as a % of DMSO control) as a function of Sovilnesib concentration. For plots in **(B)**, **(D)**, and **(F)**, each dot indicates an individual well and bars indicate mean values. Statistical results displayed from a Mann-Whitney test. P value style: < 0.05 (*), < 0.01 (**), < 0.001 (***), <0.0001 (****). n.s. indicates not significant (> 0.05). Data shown on all plots are from three independent experiments.
